# Awareness and perspectives on paediatric dysbiosis among early-career clinicians at a tertiary care hospital in Delhi, India

**DOI:** 10.1093/jacamr/dlaa072

**Published:** 2020-10-03

**Authors:** Saurav Basu, Nidhi Bhatnagar, Sahadev Santra, Anish Laul

**Affiliations:** Department of Community Medicine, Maulana Azad Medical College, 2 Bahadur Shah Zafar Marg, New Delhi, India

Sir,

The human microbiome represents the genetic material of all the microbes—including bacteria, viruses and eukaryotes—that reside in the human body and support the metabolic and immune functions that impact overall health.^1^ In contrast, dysbiosis signifies an imbalance and reduction in the diversity of the gut microbiota that increases the risk of developing chronic inflammatory diseases, obesity, diabetes, asthma and others.^2^^,^^3^ There is growing recognition that antibiotic exposure, especially during infancy and early life, can cause a microbial imbalance, which impairs long-term health, including brain development.^4^ Furthermore, antibiotic exposure, even for a short duration, may contribute to harmful effects on the human microbiome.^5^

Antibiotics are the most common drugs prescribed to children and even in developed nations with strict antibiotic stewardship nearly 30% of antibiotics prescribed to children are unwarranted.^6^ In India, the country with the largest child cohort globally, irrational prescription of antibiotics by healthcare providers, over-the-counter non-prescription sales and self-medication by parents have contributed to an alarming growth of antibiotic resistance (ABR).^7^

It is well established that combating the challenge of ABR in India requires effective antibiotic stewardship for clinicians. However, there is a lack of information as to what extent clinicians in India have an awareness of the long-term health implications of unnecessary antibiotic exposure, particularly in the paediatric age group. The objective of the present study was to assess the awareness of and perspectives on dysbiosis and antibiotic use among early-career clinicians in India. 

We conducted a cross-sectional study at a government medical college and affiliated tertiary care hospital in Delhi, India, among early-career clinicians. We included (i) medical (Bachelor of Medicine and Bachelor of Surgery, MBBS) interns who had completed medicine, paediatrics and community medicine clinical postings; and (ii) junior residents (postgraduate students) working in any clinical department.

A total of six clinical departments were selected, of which two, the Department of Paediatrics and the Department of Community Medicine, were selected purposively and four others were selected through simple random sampling. After the outpatient clinic hours, the residents and interns were contacted consecutively and invited to participate in the study. A proportional allocation sampling strategy was applied for selecting the number of participants from each department.

Data were collected using a brief self-administered questionnaire (Figure 1) for 4 months from September 2019 onwards. We analysed the data with IBM SPSS Version 25. Results were expressed in frequency and proportions for categorical variables and mean and standard deviation for continuous variables. *P *<* *0.05 was considered statistically significant.

**Figure 1.**
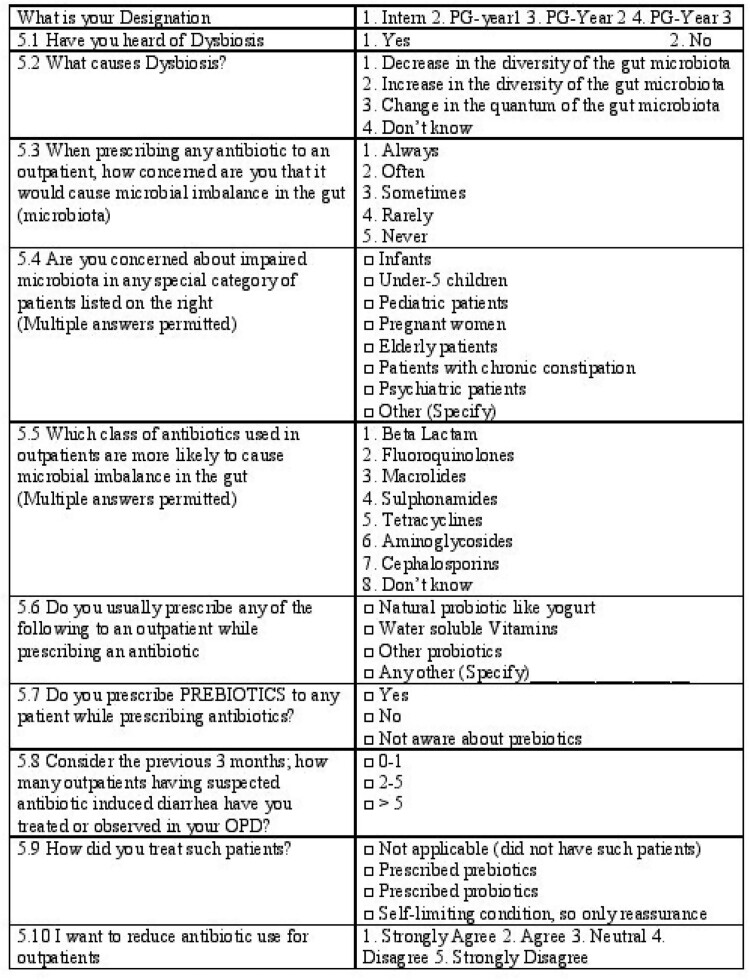
Questionnaire.

The study was approved by the Institutional Ethics Committee, Maulana Azad Medical College & Associated Hospitals, New Delhi (F.1/IEC/MAMC/68/03/2019/No/68). We obtained written and informed consent from all the study participants.

We recruited a total of 114 (57%) male and 86 (43%) female participants, including 100 interns and 100 resident doctors. Approximately three in four participants (75.4%) reported the practice of presumptive (empirical) antibiotic prescribing for their outpatients.

**Perspectives on dysbiosis influencing antibiotic prescribing behaviour of the participants:** A total of 116 (58%) participants reported having heard of the term dysbiosis. However, only 58 (29%) participants correctly reported dysbiosis being a condition signifying a decrease in the diversity of the gut microbiota.

The concern of antibiotic-induced microbial imbalance in the gut causing impaired health was moderate among the participants when estimated on a five-point Likert scale with higher scores correlating with greater concern (median = 3, mean = 3.2). However, when further probed on concerns about impaired microbiota in any special category of patients, infants (81%) and children under 5 years (82%) were considered by the participants to be the most vulnerable to antibiotic-induced adverse effects resulting from alterations in the gut microbiome.

Nearly 10% of participants were unsure whether exposure to any specific antibiotic class correlated with an increased risk of impaired microbial balance in the gut. Almost all other participants incorrectly attributed a higher risk of dysbiosis to the intake of both broad- and narrow-spectrum antibiotics.

**Practices related to control of antibiotic-induced adverse effects on the gut:** A majority (50.5%) of the participants usually prescribed curd or yoghurt as a natural probiotic during antibiotic prescribing, while 52 (26%) only prescribed water-soluble vitamins. Prebiotics that induce the growth of beneficial microorganisms were prescribed along with antibiotics by 78 (39%) participants.

Approximately one-third (35.5%) of participants reported encountering two to five cases of suspected antibiotic-induced diarrhoea in their outpatient departments during the previous 3 months, while 17% reported observing more than five such cases. The condition was managed most commonly using probiotics by 37.5% of participants, while 19% considered it a self-limiting condition requiring no specific treatment.

A strong behavioural intention to reduce antibiotic use was reported by 67 (33.5%) participants, which was significantly associated with perceived clinical concern over antibiotic-induced dysbiosis (*P *=* *0.008).

Presumptive antibiotic prescribing is inevitable in resource-constrained settings with high laboratory turnabout time, difficulties in laboratory access and high costs. The present study shows that a majority of early-career clinicians lacked awareness of the emergent threat of dysbiosis induced by antibiotic use, especially in early life. Furthermore, they did not perceive it as a significant health concern, unlike the other medical dilemmas associated with antibiotic use commonly encountered in outpatient settings.^8^ Consequently, educating and sensitizing clinicians to the evaluation of the long-term health risks against the potential benefits when considering the outpatient prescription of antibiotics to children is acutely warranted in Indian health settings. Future studies should also ascertain the effect of incorporating knowledge of dysbiosis within antibiotic stewardship programmes in promoting responsible antibiotic prescribing behaviours among clinicians.

## Funding 

This study was conducted as part of our routine work.

## Transparency declarations

None to declare.
